# Effects of Graphene Oxide Nanofilm and Chicken Embryo Muscle Extract on Muscle Progenitor Cell Differentiation and Contraction

**DOI:** 10.3390/molecules25081991

**Published:** 2020-04-23

**Authors:** Jaśmina Bałaban, Mateusz Wierzbicki, Marlena Zielińska, Jarosław Szczepaniak, Malwina Sosnowska, Karolina Daniluk, Dominik Cysewski, Piotr Koczoń, André Chwalibog, Ewa Sawosz

**Affiliations:** 1 Department of Nanobiotechnology and Experimental Ecology, Institute of Biology, Warsaw University of Life Sciences, 02-787 Warsaw, Poland; jasmina_balaban@sggw.pl (J.B.); mateusz_wierzbicki@sggw.pl (M.W.); marlena_zielinska_gorska@sggw.pl (M.Z.); jaroslaw_szczepaniak@sggw.pl (J.S.); malwina_sosnowska@sggw.pl (M.S.); karolina_daniluk@sggw.pl (K.D.); ewa_sawosz@sggw.pl (E.S.); 2 Spectrometry Laboratory, Institute of Biochemistry and Biophysics, Polish Academy of Science, 02-106 Warsaw, Poland; dominikcysewski@gmail.com; 3 Department of Chemistry, Institute of Food Sciences, Warsaw University of Life Sciences, 02-787 Warsaw, Poland; piotr_koczon@sggw.pl; 4 Department of Veterinary and Animal Sciences, University of Copenhagen, 1870 Frederiksberg, Denmark

**Keywords:** graphene oxide, in vitro, muscle contraction, myotube formation, tissue extract

## Abstract

Finding an effective muscle regeneration technique is a priority for regenerative medicine. It is known that the key factors determining tissue formation include cells, capable of proliferating and/or differentiating, a niche (surface) allowing their colonization and growth factors. The interaction between these factors, especially between the surface of the artificial niche and growth factors, is not entirely clear. Moreover, it seems that the use of a complex of complementary growth factors instead of a few strictly defined ones could increase the effectiveness of tissue maturation, including muscle tissue. In this study, we evaluated whether graphene oxide (GO) nanofilm, chicken embryo muscle extract (CEME), and GO combined with CEME would affect the differentiation and functional maturation of muscle precursor cells, as well as the ability to spontaneously contract a pseudo-tissue muscle. CEME was extracted on day 18 of embryogenesis. Muscle cells obtained from an 8-day-old chicken embryo limb bud were treated with GO and CEME. Cell morphology and differentiation were observed using different microscopy methods. Cytotoxicity and viability of cells were measured by lactate dehydrogenase and Vybrant Cell Proliferation assays. Gene expression of myogenic regulatory genes was measured by Real-Time PCR. Our results demonstrate that CEME, independent of the culture surface, was the main factor influencing the intense differentiation of muscle progenitor cells. The present results, for the first time, clearly demonstrated that the cultured tissue-like structure was capable of inducing contractions without externally applied impulses. It has been indicated that a small amount of CEME in media (about 1%) allows the culture of pseudo-tissue muscle capable of spontaneous contraction. The study showed that the graphene oxide may be used as a niche for differentiating muscle cells, but the decisive influence on the maturation of muscle tissue, especially muscle contractions, depends on the complexity of the applied growth factors.

## 1. Introduction

Tissue engineering is hugely challenging but offers hope for better therapeutic processes after traumatic muscle injury, tumor ablation, or muscle disease [1]. There is a pressing need within regenerative medicine to find an effective muscle regeneration technique. Stem cells, growth factors, and artificial niches are recognized as fundamental elements for tissue engineering [2]. The interaction between these factors is not entirely clear. Furthermore, the use of a complex of complementary growth factors instead of a few strictly defined ones could increase the effectiveness of muscle tissue maturation. In our research, we used chicken embryo muscle precursor cells; chicken embryo muscle extract (CEME), containing a cocktail of growth factors; and graphene oxide (GO) nanofilm as a potential niche.

### 1.1. Muscle Precursor Cells

Skeletal muscles are derived from somites, which are formed from the mesoderm. Somites give rise to the dermomyotome, myotome, and sclerotome [3]. The cells with myogenic potential are known as myoblasts, and after they exit the cell cycle, a multinucleated myotube and myofiber form by fusion [4]. The process of progenitor muscle cell differentiation is controlled by myogenic regulatory factors (MRFs)—a group of transcription factors comprising Myf5, MyoD, MyoG, and MRF4. MRFs create a network of auto- and cross-regulatory interactions between factors, which modulate expression levels [5]. With the exception of Myf5, each factor can individually activate the differentiation of muscle progenitor cells [6], while Myf5 and MyoD regulate cell proliferation. MyoD is also associated with the promotion of the myogenic determination process, whereas MyoG is crucial for the terminal maturation of myoblasts, and inhibition of MyoG expression results in a loss of skeletal muscle [7]. The paired-box transcription factors, Pax3 and Pax7, play an important role in early myogenesis, and are specific for dermomyotome-derived muscle progenitor cells [8,9]. After activation of MRFs, Pax3 expression is downregulated [10]. Expression of Pax7 is also distinctive for muscle satellite cells in mature tissue [11].

### 1.2. Growth Factors

Tissue and embryo extracts have been commonly used in cell cultures in vitro. Skeletal muscle extract applied to a motoneuron culture enhanced cell survival [12,13] and induced differentiation in vitro [14]. Extracts from a chicken embryo and chicken embryo eye ensured the survival of embryo ciliary ganglionic neurons [15]. Chicken embryo extract is widely used as a growth-promoting factor, as it enhances the proliferation of myogenic cells and affects the timing of the final differentiation [16,17,18]. It can, therefore, be assumed that the composition of growth factors contained in the muscle of the embryo, just before hatching, when the body focuses on the preparation of the musculoskeletal system (leg muscles) for intensive work after hatching, will contain a set of proteins necessary to activate spontaneous contractions. In the culture of muscle cells, natural substances that stimulate proliferation or differentiation, such as horse serum [19] or whole embryo extract [16,17,18,20], are used, but the extract from the embryo muscle taken at the end of embryogenesis has not been previously used in muscle cell culture.

### 1.3. Niches

Carbon-based nanomaterials are promising substrates for reinforcing artificial niches and mimicking native muscle extracellular matrix [21]. The presence of oxygen-containing groups on the carbon sheet surface makes GO a non-toxic and biocompatible material [22]. However, the other physicochemical features of GO can significantly modify its biocompatibility [23,24]. In our studies on rats, we documented the lack of toxicity of high doses of GO administered into the peritoneum [25,26]. Other authors, although they demonstrated the location of GO in various tissues, especially in the lungs, nevertheless, pointed to its relative biocompatibility [27].

Moreover, the physical properties of GO, such as its roughness, surface topography, thickness, elasticity, and hydrophilicity, can modulate cell behavior and the differentiation process via mechanical or chemical signaling [28]. Thus, GO can provide authentic extra-cellular stimuli for muscle progenitor cells. However, this requires GO is kept outside of the cell as part of the matrix.

Many studies have demonstrated the effects of GO on proliferation, adhesion, and the differentiation of various types of cells [29]. GO enhances the expression of the gene involved in cardiomyogenic differentiation of human embryonic stem cells in vitro [30]. The application of the GO complex with a conductive polymer improved neural cell in vitro differentiation [31]. GO implanted together with chitosan or polylactic acid scaffold were an excellent, biocompatible matrix for bone mineralization in the process of their regeneration [32,33]. In in vivo studies with the nanocomposite containing 1% GO, introduced as a subcutaneous implant to rats, it was biocompatible, and interestingly, exhibited antibacterial properties [34].

GO encapsulated in alginate increased the viability of C2C12 cells [35]. A combination of peptide-decorated polylactic glycolic acid (PLGA) nanofibers and GO stimulated the differentiation of C2C12 cells [36], and a nanocomposite of polycaprolactone (PCL) with GO promoted adhesion and proliferation. Fibroblasts cultured on GO film showed different expression profiles for a focal adhesion protein, indicating a high affinity of the cells to the GO substrate [37]. A study with a gelatin-GO nanocomposite demonstrated an improvement in the differentiation process in C2C12 cells by scaffold-cell interactions only [38], and a similar result was obtained with a PCL-GO scaffold [39]. 

The aim of this study was to determine whether the use of muscle extract obtained from chicken embryos prior to hatching would affect the differentiation and functional maturation of muscle precursor cells and the ability of potential pseudo-tissue muscle to spontaneously contract. The study used two types of surfaces to perform in vitro culture: standard polystyrene culture plates and plates covered with GO nanofilm, mimicking a biocompatible niche.

## 2. Results

### 2.1. GO Characterization

**Figure 1:**
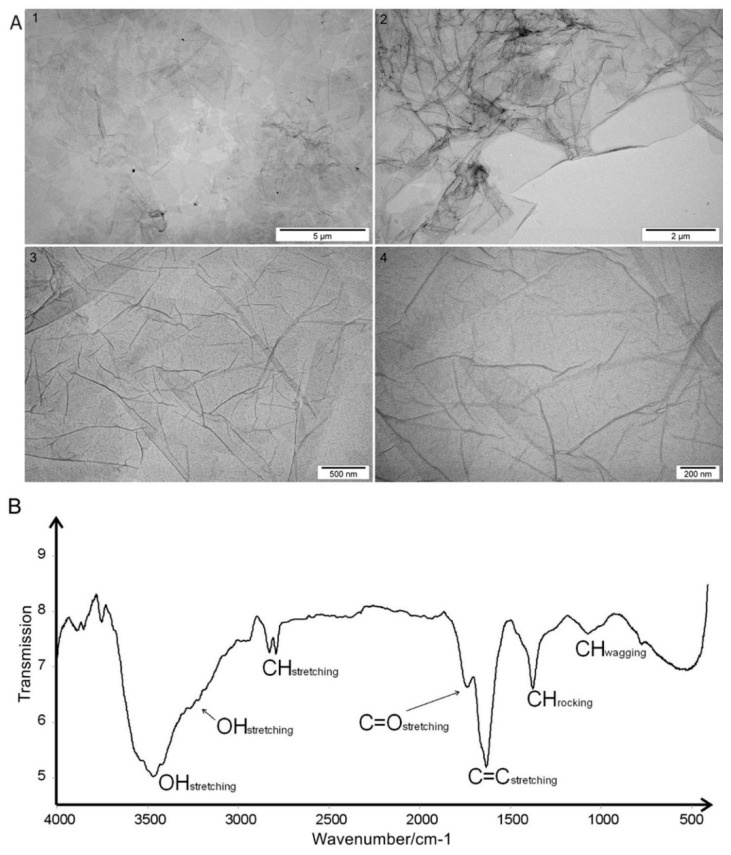
(**A**) Transmission electron microscopic images of graphene oxide flakes at increasing magnification: 1) 5,000, 2) 8,500, 3) 34,000, 4) 70,000; (**B**) Fourier Transform Infrared Spectroscopic spectrum of graphene oxide with the assignment of bands to appropriate vibrations of groups and bonds present in the sample.

GO was purchased in the form of an aqueous solution with high stability, a dark brown color, and a concentration of 4 mg/mL No tendency for GO to agglomerate or precipitate was observed after 6 months. The zeta potential of GO suspended in water at a concentration of 1 mg/mL was 27.1 mV. Transmission Electron Microscopy (TEM) imaging was used to assess the morphology of the flakes of GO (Figure 1A). After drying, GO formed flakes one to three layers thick and 2 to 4 μm in size. 

Fourier Transform Infrared Spectroscopy (FT-IR) analysis revealed a broad, very intense, structured band that was located at 3457 cm^−1^ with shoulder maximum at 3217 cm^−1^ in the graphene IR spectrum. There were two bands generated by C-H stretching at 2817 and 2779 cm^−1^. The region at 1800–1500 cm^−1^ contained two distinct bands generated by C=O stretching (1728 cm^−1^) and C=C ring stretching (1621 cm^−1^). Bending vibrations generally located at a lower energy in the fingerprint spectral region were at 1367 and 1061 cm^−1^ (Figure 1B).

### 2.2. GO Nanofilm Characterization

**Figure 2:**
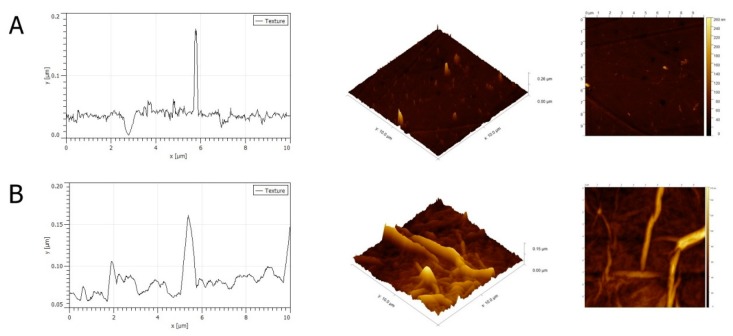
Atomic Force Microscopy (AFM) mages and a topography model of the surface of a culture plate (**A**) and graphene oxide nanofilm (**B**).

An aqueous GO solution with a concentration of 1 mg/mL was used to prepare the nanofilm for in vitro studies. Atomic Force Microscopy (AFM) analysis of GO as a nanofilm on the bottom of a culture-plate well was performed. Numerous sharp peaks were present on the surface of the culture plate (Figure 2A). GO nanofilm caused a decrease in the average roughness of the culture plate surface. The average roughness of GO nanofilm was 1.5 to 2.1 nm, whereas the roughness of the standard polystyrene culture plate was 7.2 nm. The topography of the nanofilm was irregular and crested (Figure 2B). The surface coated with GO nanofilm was more aligned and the peaks were rounded and less jagged compared to the surface of the culture plate.

### 2.3. Chicken Embryo Muscle Extract Analysis

The mass spectrometry analysis of muscle samples identified 1470 proteins and 6893 unique peptides (Table S2), which are available in the PRIDE repository under PXD015146. In preliminary studies, which allowed the emergence of spectacular pro-contraction activity of CEME, various substances and extracts were studied, which were a cocktail of growth factors, including horse serum, embryo brain extract, embryo extract, or liver extract. The spontaneous contraction was observed only under the influence of CEME, none of the mentioned substances had the effect on contractions of embryonic muscle cells. Consequently, further morphological and molecular studies were conducted using a functionally effective factor, which CEME proved to be.

However, in order to identify the potential proteins responsible for muscle cell contraction, the proteins common to CEME and liver extract were rejected, resulting in the number of 249 protein candidates for top proteins in the muscle cell contraction process. Table 1 presents 59 selected top proteins associated with muscle contractions to the greatest extent. 

Proteins specific only for CEME were selected and grouped according to the relationship of their function to extracellular matrix (ECM) components, cell structure and communication, and contraction phenomenon and enzymes related to muscle cell energy metabolism (Table 1). These proteins were selected as potential stimulators of muscle progenitor cell differentiation and maturation. 

### 2.4. Assessment of Proper Concentration of Chicken Embryo Muscle Extract Supplement for in Vitro Experiment

To assess the optimum concentration of extract added to the culture media, a Trypan Blue assay was performed. The number of live cells after 48 h of culture with different concentrations of extract (5%, 2.5%, and 1%) was measured. In the control group, the viability was 75.5%. In the group with 5% extract, the viability was 42%. A decrease in the concentration of extract resulted in increased the live cell count. In the groups with 2.5% and 1% extract addition to the culture media, cell viabilities of 62% and 82.5% were observed, respectively. The viability of cells with 1% supplementation of CEME was the highest. Consequently, in further experiments 1% CEME addition was applied. 

### 2.5. Cell Morphology

**Figure 3:**
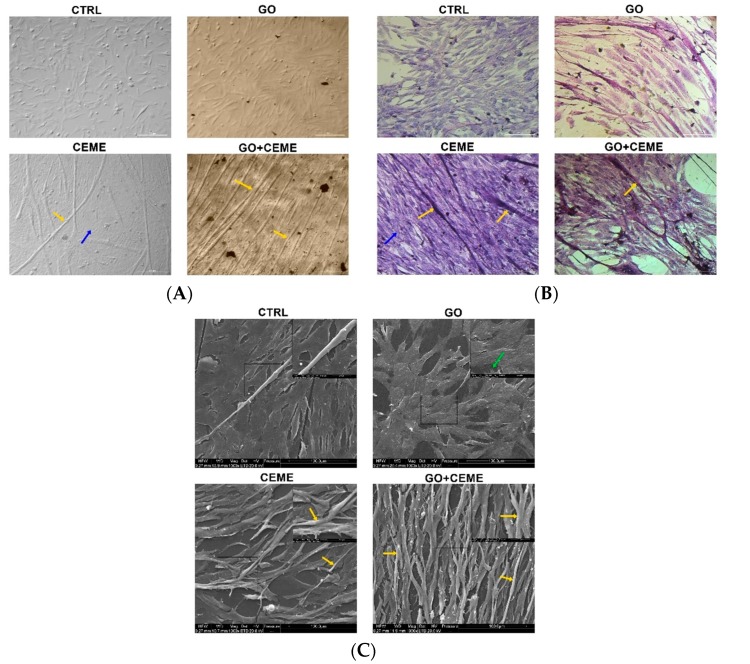
Cell morphology evaluated by optical microscopy (**A**,**B**) and scanning electron microscopy (**C**); the images show the control group (CTRL), cells cultured on graphene oxide nanofilm (GO), cells cultured with the addition of the extract (CEME), and cells cultured on GO nanofilm with addition of the extract (GO + CEME); cells were stained with eosin/hematoxylin for visualization of nucleic acids and proteins (**B**); myotubes (yellow arrows), filopodia (green arrow); multilayer of undifferentiated cells (blue arrows).

**Figure 4:**
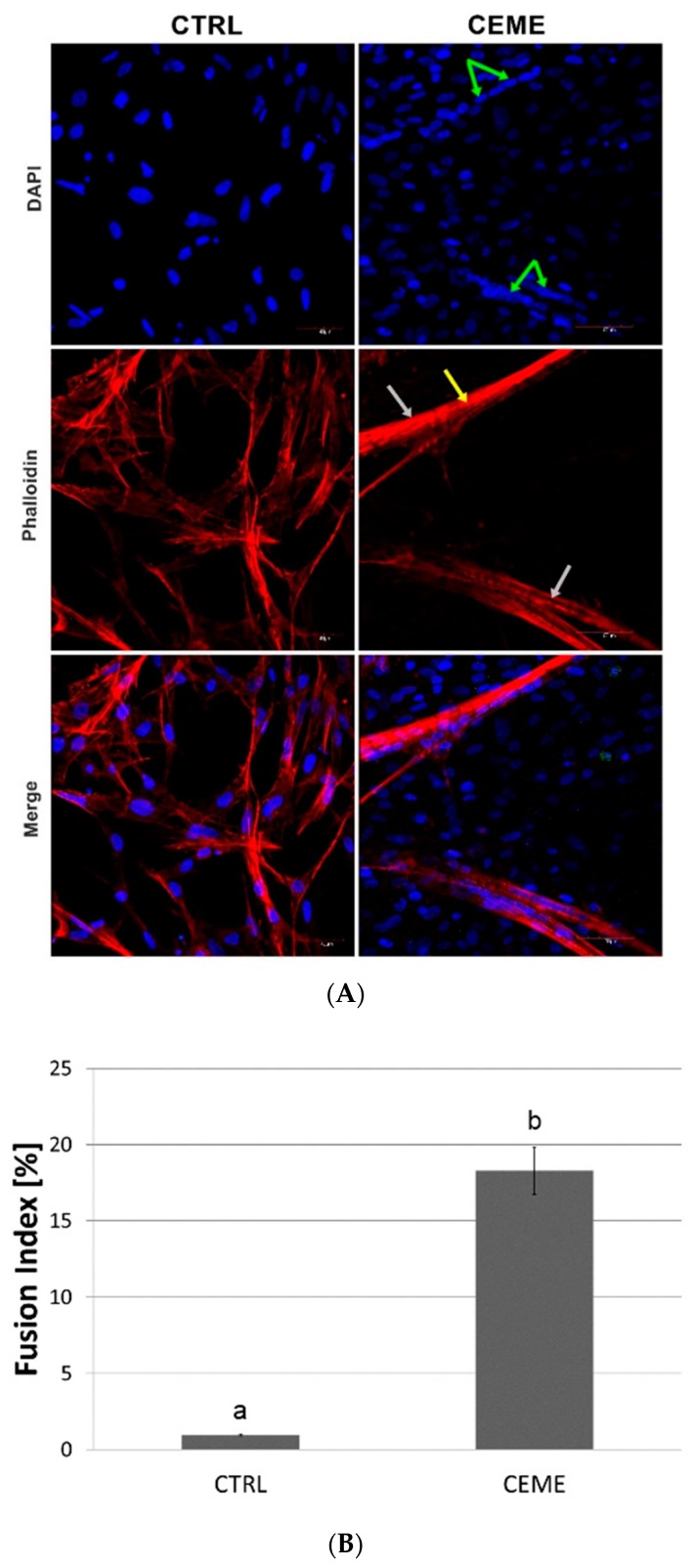
(**A**) Fluorescent images with labeled nuclei (blue) and actin (red) of cells after 5 days of culture: control group (CTRL); culture with chicken embryo muscle extract supplementation (CEME); nuclei in myotubes (green arrows), myotubes (grey arrows), striated sarcomeric structure (yellow arrow); quantitative analysis of the fusion index of the differentiating cells (**B**). The error bars represent standard deviations. Different letters (a, b) above the columns indicate statistically significant differences between the groups (*p* ≤ 0.05).

The microscopic images of the experimental cultured cells show several morphological changes compared to the control group (Figure 3). Cells cultured on the GO nanofilm were strongly attached to the substrate; they were more stretched and flatter with numerous filopodia (Figure 3C, [GO]), and the cell surfaces were gently corrugated. Cells cultured with the extract formed several dense layers, and there were numerous long, branched, multinucleated myotubes at the top (Figure 3A, [CEME]). The myotubes were positioned in an unorganized manner, crossing at various angles (Figure 3A, [CEME]). Myotubes were often longer than 1000 μm and were 10–20 μm wide. Fluorescent images revealed the striated structure of the myotubes and confirmed the presence of multinucleated muscle cells (Figure 4A, [CEME]). Fusion index increased significantly in cultures with CEME supplementation, compared to that in the control group (Figure 4B). The combination of the GO nanofilm with the extract induced the presence of an undifferentiated multilayer of cells with multinucleated myotubes on the top. The myotubes on GO nanofilms were positioned in a more organized manner (Figure 3A, [GO]).

The key result was the spontaneous contraction activity of myotubes in the cultures with extract supplementation (Video S1). One to three contractions per minute were noted, an average of 1.7 contraction per minute. Rhythmic contractions of primary muscle fibers were observed under the influence of CEME, both in the group without the GO nanofilm as well as with the GO nanofilm. Moreover, there was no difference in contractions between the cells of the CEME and GO + CEME groups. There were no contractions in the control and GO groups. Thus, only the growth factor cocktail (CEME), not the substrate characteristics, generated the physiological activity of cells involved in myogenesis.

### 2.6. Cytotoxicity and Viability of Muscle Cells

**Figure 5:**
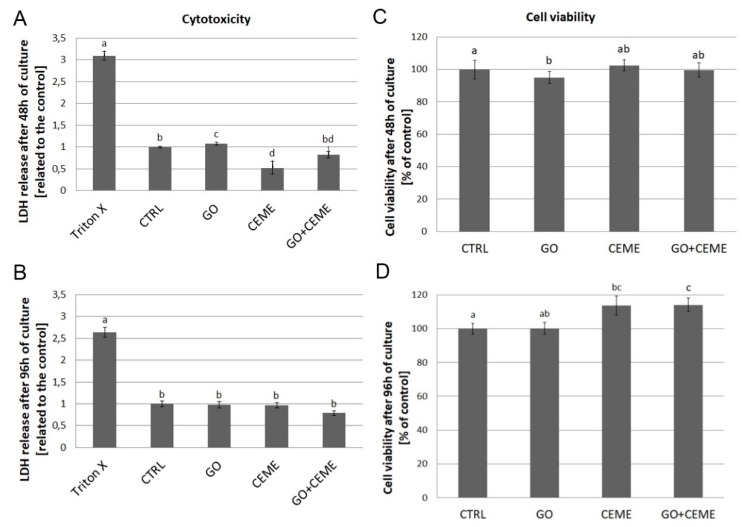
Lactate dehydrogenase (LDH) release (**A**,**B**) and cell viability (**C**,**D**) were determined using LDH and MTT assays, respectively. Tests were performed after 48 and 96 h of primary culture. Negative control for LDH maximum release (Triton X), control group (CTRL), cells cultured on graphene oxide nanofilm (GO), cells cultured with addition of the extract (CEME), and cells cultured on GO nanofilm with addition of the extract (GO + CEME). The error bars represent standard deviations. Different letters (a, b, c, d) above the columns indicate statistically significant differences between the groups (*p* ≤ 0.05).

To evaluate the cytotoxicity of GO nanofilm and the addition of the extract, the lactate dehydrogenase (LDH) assay was performed after 48 and 96 h of culture (Figure 5A,B). The test was based on the enzymatic reduction of NAD^+^ by LDH released from damaged cells into the culture media. LDH levels reflect the integrity of the cell membrane. Compared to the control group, the presence of GO nanofilm slightly elevated LDH release from cells after 48 h of culture, but there were no significant differences after 96 h. 

To compare the effect of GO nanofilm and CEME on cell viability, the ability of cells to reduce tetrazolium salt (MTT) and produce insoluble formazan crystals was tested. MTT reduction was measured after 48 and 96 h of culture (Figure 5C,D). Compared to the control group, GO nanofilm slightly affected cell viability but the differences were negligible. 

### 2.7. Expression of Genes

**Figure 6:**
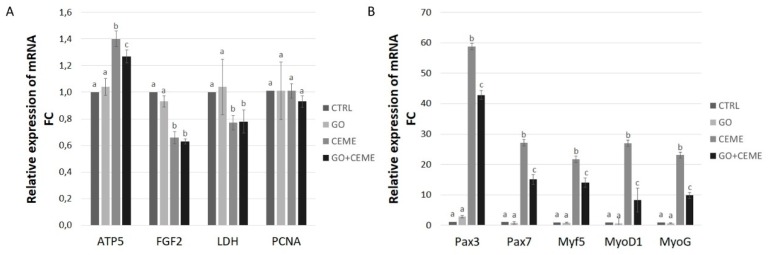
Real-Time PCR analysis of gene expression at the mRNA level in muscle progenitor cells from the chicken embryo after 5 days of primary culture. Expression of genes related to proliferation, basic metabolism (**A**), and muscle cells differentiation (**B**) was investigated; the figure shows the results for the control group (**CTRL**), cells cultured on GO nanofilm (GO), cells cultured with addition of the extract (CEME), and cells cultured on GO nanofilm and extract (GO + CEME); Relative expression was calculated using housekeeping genes, ACTB and GAPDH; the results are presented as 2-ΔΔCT values compared to the control group; different letters above the columns indicate statistically significant differences between the groups (*p* ≤ 0.05). The error bars represent standard deviations.

Changes in gene expression at the mRNA level were examined using the Real-Time PCR method. Compared to the control group, GO nanofilm had no significant effect on the expression of genes related to basic metabolism and proliferation—*ATP5B*, *FGF2*, *LDH5*, and *PCNA*. The extract supplementation resulted in a significantly increased level of *ATP5B* gene expression, whereas *FGF2* and *LDH5* gene expression was downregulated in comparison to the control. *PCNA* gene expression was not influenced by any factor. The combined presence of the GO nanofilm and the CEME had a significant positive effect on *ATP5B* gene expression. *FGF2* and *LDH5* genes were downregulated in comparison to the control group, which is similar to the results obtained with the addition of extract only (Figure 6A). 

Compared to the control group, the presence of GO nanofilm had no significant effect on expression at the mRNA level for all genes associated with the differentiation process-*Myf5*, *MyoD1*, *MyoG*, or *Pax3* and *Pax7* genes. The addition of extract to the culture media significantly increased the level of mRNA of *Myf5*, *MyoD1*, *MyoG*, *Pax3*, and *Pax7*. The expression profile of cells cultured on GO nanofilm with the addition of extract was also upregulated in all investigated genes; however, the fold change was lower than that in the extract-only group (Figure 6B).

## 3. Discussion

Physiologically fully mature muscle cells that form muscle-like tissue through muscle fiber cooperation should be capable of spontaneous contractions. Previous research has demonstrated that it is possible to create muscle-like tissue that is capable of twitching under the influence of externally applied electrical impulses [22,40,41]. The study presented here is the first to document the generation of in vitro muscle cell contraction using only selected growth and regulatory factors. Furthermore, we found that this effect did not depend on the physicochemical characteristics of the cell culture surface but solely on the availability of protein molecules, i.e., growth factors and their regulators. To verify our findings, we used two variables for in vitro culture: a differentiated surface and a differentiated medium. 

The surface of the in vitro culture plate was modified by covering with a thin layer of GO. When the surface of the plate and the GO layer were compared, the GO layer presented a smoother surface with a lower density of wrinkles and moderate roughness [42]. However, the roughness of GO depends on many factors and is likely to be variable [43,44]. Using a thin layer of GO, the carbonaceous biocompatible material can be coated onto the surface of an in vitro culture vessel, and this has proven to be a reliable, repeatable, and sterile method. 

In general, cells maintained on GO nanofilm exhibit normal muscle cell and muscle-cell precursor morphology. However, we observed more stretched and wrinkled cell surfaces and numerous protrusions, which could be related to the structure of the GO nanofilm [29] or to the surface chemistry. 

A toxic effect and the reduction of proliferation of GO cultured cells were observed only after 48 h. After 96 h of incubation, no harmful effect on GO surface was observed. This phenomenon may have resulted from the interaction between cells and GO, and as a result of depositing on the GO surface proteins secreted by muscle precursor cells and by the optimization of the niche conducive to colonization. The lack of a negative GO effect on muscle and myogenic cells is also confirmed by other studies [39,44,45,46,47].

The presence of GO nanofilm in cell culture did not affect *LDH* or *ATP5B* gene expression at the mRNA level. These basic markers of metabolism, particularly oxygen and anaerobic metabolism, indicate that the GO nanofilm is neutral to muscle cells and precursors despite the presence of oxygen groups on the surface. However, in a study on the MFC-10A cancer cell line, the presence of PEG-modified GO nanosheets downregulated the expression of proteins involved in the oxidative phosphorylation, but this effect was not observed in non-cancerous cells [48]. Furthermore, our research demonstrated that the presence of GO nanofilm has no effect on *MyoD1*, *Myf 5*, *MyoG*, *Pax3*, and *Pax7* gene expression. Expression of these genes is an important determinant of myogenesis—the basic role of muscle cells and their precursors. This indicates that GO biological activity is probably influenced by the type of cells. 

The second experimental factor was the modification of the culture medium. CEME was used as an additive to improve the standard medium (DMEM). We expected the extract from an 18-day-old chicken embryo hind limb to complement the stimulation of muscle progenitor cell differentiation and physiological maturation. This speculation was based on the fact that the extract contained proteins involved in the differentiation of muscle cells, which is at a very advanced stage on the 18th day of embryonic development. Furthermore, the extract contained proteins produced by muscle cells and their precursors, as well as all other cells present in the developing muscle tissue that cooperate with the muscle cells. The extract contained proteins involved in preparing the muscles for intense work, which must occur just before and immediately after hatching, and these proteins are released by muscle cells particularly [49]. In addition, muscle cells secrete hundreds of proteins and peptides upon contraction [50].

The addition of the extract strongly affected chicken muscle progenitor cell morphology. A significantly higher fusion index was noted. We observed elongated, multinucleated, branched, and striated myotubes, which exhibit spontaneous contractile activity, and these features are typical of differentiated cell morphology [29,51,52]. These results clearly show that the differentiation process occurred and that the addition of extract to the culture media led to the formation of a tissue-like structure of muscle cells and their precursors. However, the resulting myostructure exhibited two layers: in vivo-like muscle fibers and flat, round, mononuclear cells lining the bottom of the culture vessel. These cells probably had myogenic potential, as was seen when an undifferentiated subpopulation of cells was added to a primary culture of rat muscle cells [53].

The observation of cell morphology has been confirmed by cytotoxicity and viability results. The LDH release from cells did not decease, while the addition of extract significantly increased the number of viable muscle cells and their precursors after 96 h of culture. This was also noted after treatment of a skeletal muscle cell culture with extract that was obtained from 12-day-old chicken embryo brain and liver [54]. In a muscle cell culture obtained from 10-, 11-, and 12-day-old chicken embryos, the addition of chicken embryo extract showed a stimulatory effect on proliferation and differentiation [17,55]. Moreover, the addition of chicken embryo extract in combination with high serum content in the culture media enhanced the proliferation and growth of murine myoblasts [56].

In cells maintained in culture media with CEME, *Pax7* expression was strongly upregulated. *Pax7* is a key factor in satellite cell myogenic determination. Deletion of the *Pax7* gene resulted in a significant decrease [57] or complete elimination of satellite cell populations [58], but inactivation of the *Pax7* gene did not affect adult myogenesis [48]. An analysis of the expression profile of *Pax3* and *Pax7* in rat muscle cells indicated that the level of *Pax7* gene expression decreased during the maturation of myoblasts and formation of myotubes, contrary to our observation that *Pax7* was stimulated in parallel with the up-regulation of the genes responsible for differentiation. In an experiment with rat muscle cells, a subpopulation of undifferentiating, proliferating cells with *Pax3* and *Pax7* expression was also observed [58]. This phenomenon could explain the high expression of *Pax3* and *Pax7*, which could be related to the dense multilayer of undifferentiated cells that were observed on the bottom of the culture plate and became a renewable source of differentiating myoblasts. This would explain the simultaneous and strong increase in the expression of genes responsible for the differentiation of muscle cells and their precursors into muscle fibers, together with the self-renewal of the pool of cells that are the early precursors of myocytes.

The addition of CEME to the culture media has a strong effect on MFRs expression. The results obtained in this study demonstrated that supplementation of the culture media with the extract enhanced the myogenic differentiation of chicken muscle progenitor cells. We observed a significant up-regulation of expression of all investigated MRFs (*Myf5*, *MyoD*, and *MyoG*) in cells maintained in media with CEME. MRFs are critical factors in the maturation of muscle cells [59,60]. Moreover, the expression of *FGF2*, which is considered to be a stimulator of proliferation and a strong inhibitor of differentiation [61], was downregulated in cells cultured with the extract. From a molecular point of view, downregulation of FGF should be coupled with downregulation of Pax 7, however, in the case of heterogeneous culture, which is the primary culture of cells taken from the embryo muscle, expression of mRNA is a resultant expression of the sum of different cells. It should also be taken into account that the chicken embryo muscle undergoes very strong myogenesis just before hatching, but also it contains over 30% of satellite cells [52], whose proteome may promote the proliferation of satellite cells in the culture. Therefore, primary culture, which on the one hand, well reproduces the natural conditions of myogenesis, on the other hand, creates difficulties in interpreting the results. It can certainly be stated that a precise explanation of the above mechanisms requires wider and more precise research.

Differentiation of myoblast cells and their precursors into mature muscle fibers was also confirmed by the change in cellular metabolism from anaerobic processes to the predominance of oxidative phosphorylation. *LDH* gene expression decreased and *ATP5B* gene expression simultaneously increased in cells that were cultured with the extract. The results are in agreement with changes occurring in muscle cells during differentiation, when a high glycolytic state in the myoblasts transforms into intensive oxidative phosphorylation in mature muscle cells [62]. 

The morphology and molecular status of differentiating cells, as well as their metabolism profile, undoubtedly indicate the formation of a structure containing mature muscle cells under the influence of CEME proteins. However, equally important evidence for the physiological maturity of the structure is its ability to undergo spontaneous contractions. The present results, for the first time, clearly demonstrated that the cultured a tissue-like structure was capable of inducing contractions that were not caused by external factors. 

Use of the embryonic muscle extract affected the physiological ability of the muscle cells to contract, regardless of the type of surface (with or without GO) used. The extract contained proteins derived from various muscle-related tissues, including nervous tissue, connective tissue, blood vessels, and blood, as well as various structural proteins, such as of the ECM, cytoplasm, contractile proteins, and neural structures and their regulators, e.g., metabolic enzymes. These proteins acted as agents to directly stimulate muscle contractions or as factors to indirectly create an optimal contraction environment. 

ECM proteins, especially collagen alpha 1 and alpha 3, decorin, laminin, and fibromodulin, can affect ECM maturation and cooperation with muscle cells. Laminin controls the structural integrity of muscle tissue and the connection of the intracellular actin structure with ECM [63,64,65], and fibromodulin is a regulatory factor for myoblast differentiation [66]. 

The extract was also the source of many proteins responsible for ECM–cell–cytoskeleton interactions. Some proteins form important links with actin-containing filaments of the cytoskeleton [67,68]. However, it seems that the key proteins are those that directly participate in muscle contraction, i.e., motor proteins and their regulators (myosin light chain, myosin-1B, myosin regulatory light chain 2, myosin light chain kinase). Tropomyosin alpha-1 chain–actin-binding protein cooperates with troponin C and is involved in the contractile system of vertebrate striated muscles. Troponin C and tropomyosin form thin fibers, which act to bind Ca^2+^ during the contraction process [69]. Calcium-calmodulin-dependent protein kinase II with mitogen-activated protein kinase can activate myosin light chain kinase [67]. In addition, it can be assumed that the proteins responsible for the management of Ca ions during muscle contractions activate this process in vitro. Calsequestrin (a calcium-binding protein responsible for Ca homeostasis after muscle contraction) supports the storage of a large amount of Ca ions within the sarcoplasmic reticulum. Before birth, the levels of calsequestrin in the muscles increase rapidly [68], indicating that it also stimulates muscle physiological maturation. 

Furthermore, CEME components are associated with the nervous system and neural/neuromuscular communication and could be the key to spontaneous muscle cell contraction in the absence of electrical impulses. A recent study revealed that neuromodulin (specific to the nervous system) is present in myoblasts, differentiating muscle cells, and mature muscle fibers [70]. Neural cell adhesion molecule is associated with the fusion of myoblasts, and its level increases during myoblast differentiation. Agrin is a protein critical to neuromuscular junction development and maintenance [71], and utrophin is present in neuromuscular and myotendinous junctions and vasculature [72]. The administration of proteins involved in the cooperation of muscle cells and nerves, especially signal induction, may have been important for the activation and duration of the contractions.

Muscle contractions also require energy; therefore, the proteins involved in ATP metabolism would, undoubtedly, facilitate contraction mechanisms. The presence of ATP synthase subunit gamma, responsible for producing ATP from ADP, is fundamental. In addition, creatine kinase (CK), which catalyzes the transfer of a phosphate group from phosphocreatine to ADP to regenerate ATP, is a key enzyme for cellular ATP homeostasis. Interestingly, the CEME provided a source of both muscle- and brain-specific forms of CK. 

Finally, it has to be underlined that the present measurements elucidated the potential effects of a variety of proteins and cells from CEME cultivated on GO surface, thereby including interactions in-between released proteins. However, a further step is necessary to identify groups of key proteins directly affecting tissue development by interacting in a heterogeneous environment of muscle tissue. This quantification might help to develop the solutions for future clinical therapy.

## 4. Materials and Methods

### 4.1. Characterization of Graphene Oxide and Preparation of a Graphene Oxide Nanofilm

GO water solution (4 mg/mL) was purchased from NanoPoz (Poznań, Poland), and GO was produced by a modified Hummers’ method. The obtained GO flakes had diameters of between 2 and 30 μm, an average size of ~4 μm, and contained 36% oxygen (manufacturer’s data). Chemical characterization of GO was performed with FT-IR. Spectra were registered with the use of a Perkin Elmer System 2000. The roughness of GO was measured by AFM, and imaging was performed on an MFP 3D BioAFM with the commercial triangular cantilever (MPLCT, Bruker, Camarillo, CA, USA), spring constant k = 0.10 N/m in AC mode. The measurements were performed on 10 × 10 μm areas, in ten places. The shape and size of GO films were examined using a TEM - JEM-1220 (JEOL, Tokyo, Japan) at 80 kV and a TEM CCD Morada 11 megapixels camera (Olympus Soft Imaging Solutions, Munster, Germany). The zeta potential and size of GO in solution were measured by light scattering using a ZetaSizer Nano ZS model ZEN3500 (Malvern Instruments, Malvern, UK). All measurements were repeated three times. 

Experimental GO nanofilm was prepared by applying a GO solution to the bottom of a well in a culture plate. GO was air dried in a laminar chamber. For nanofilm preparation, a concentration of 1 mg/mL was used. The GO solution was sonicated for 30 min before the experiment. Then, under sterile conditions, the GO solution was embedded onto the bottom of the culture plate (40 μg) and dried at room temperature in a laminar chamber. As a result of the self-assembly and drying process, the plastic bottom of the well was covered with a thin, strongly adhesive nanofilm of GO. The prepared plates were used for further experiments.

### 4.2. Chicken Embryo Muscle Extract Preparation

Fertilized chicken (Ross 308) eggs were purchased from a certificated hatchery, sterilized in KMnO_4_ solution, irradiated with UV for 45 sec, and incubated at 37 °C and ~60% relative humidity. The tissue sample was obtained from an 18-day-old chicken embryo. The egg was removed from the incubator and slightly opened, and the embryo was pulled from the egg and decapitated. Then the explant from the hind limb was dissected, and the skin, bones, and shank were removed from the muscle tissue sample. A sample was then suspended in ultrapure water and homogenized with frozen metal balls in a TissueLyser ball mill (Qiagen, New York, NY, USA). The homogenate was centrifuged at 1400× *g* for 45 min at 4 °C. The supernatant was transferred into a new tube and stored at −80 °C. In order to reject the proteins contained in CEME, which are not involved in the activation of muscle contraction, an extract of chicken embryo liver was also prepared according to the method described above.

The protein composition of the extract was analyzed by mass spectrometry (MS) using an Orbitrap Elite (Thermo Fisher Scientific, Massachusetts, MA, USA) connected to a Water Acquity HPLC. Protein extract (150 μL) was concentrated on Vivacon 500, 10,000 MWCO Hydrosart filters by centrifuging at 10,000× *g* for 30 min at 4 °C. Filters were washed twice with washing buffer (200 μL, 8 M urea in 100 mM NH_4_HCO_3_ aqueous solution) and centrifuged at 10,000× *g* for 20 min at 4 °C. Accordingly, the proteins were reduced by the addition of washing buffer (50 μL) with 20 mM TCEP and incubated for 30 min at room temperature, then centrifuged at 10,000× *g* for 20 min at 20 °C. Proteins were alkylated with 20 mM IAA (50 μL) in washing buffer. Digestion was performed with a protease mix, LysC/Tryp V507A (Promega, Wisconsin, WI, USA). The protease mix was resuspended in 8M urea in 100 mM NH_4_HCO_3_ (80 μL). A 40 μL aliquot of the solution was added to a filter and incubated for 3 h at 37 °C; then NH_4_HCO_3_ (400 μL) was added and the samples were incubated overnight at 37 °C. Probes were centrifuged at 10,000× *g* for 30 min at 30 °C. NH_4_HCO_3_ (100 μL) was added and centrifuged at 10,000× *g* for 30 min at 30 °C. The solution was acidified with TFA to a final concentration of 0.4% and dried with a speed-vac. The peptide mixture was fractionated using a high pH protocol on an Oasis HLB 10 mg cartridge (Waters 186000383). Cartridges were activated by washing with methanol and equilibrated with 25 mM ammonium formate at pH 10. The protein pellet was resuspended in 400 μL of 25 mM ammonium formate at pH 10 and loaded onto a cartridge. Samples were washed once with loading buffer. The elution was carried out with an increasing gradient of acetonitrile in loading buffer (5/10/15/20/30/35/40/85). The last elution was carried out with pure methanol. All fractions were dried with a speed-vac. Pellets were resuspended in 60 μL of 0.1% TFA with 2% MeCN. MS analysis was performed by LC-MS in the Laboratory of Mass Spectrometry (IBB PAS, Warsaw) [73]. Data were analyzed on the Max-Quant 1.6.3.4 platform [74,75]. The *Gallus gallus* reference proteome database from UniProt (containing 29474 protein IDs) was used. Data were deposited in the PRIDE repository under PXD015146. To analyze the function of the proteins contained in CEME, the composition of CEME proteins and the composition of liver extract proteins were compared, and then for further interpretation, all proteins common to CEME and liver extract were rejected from the CEME proteome. In this way, proteins that were not involved in the activation of contraction were eliminated, and only those that were potentially responsible for muscle contraction were left. This procedure was authorized by a preliminary study, where the lack of effect of the liver extract on muscle cell contraction was documented.

### 4.3. Primary Cell Cultures of Muscle Progenitor Cells

Fertilized chicken (Ross 308) eggs were incubated in accordance with the method described in the section *“Chicken embryo muscle extract preparation”*. Mesenchymal muscle precursor cells were obtained from an 8-day-old chicken embryo limb bud by dissection of the hind limb. The sample was inserted into tubes with trypsin for 24 h at 4 °C. The enzyme was then neutralized with culture medium, the sample was disintegrated by gentle pipetting through the tips, and cells were seeded onto a culture plate. Cells were maintained in Dulbecco’s Modified Eagle’s medium (DMEM) (Life Technologies, Texas, TX, USA) supplemented with 10% fetal bovine serum (Life Technologies, Texas, TX, USA) and 1% penicillin/streptomycin (Life Technologies, Texas, TX, USA) at 37 °C in a humidified atmosphere of 5% CO_2_/95% air in a Memmert ICO150med Incubator (Memmert, Schwabach, Germany). Cells were divided into four groups and cultured in standard conditions (CTRL), on the GO nanofilm (GO), with the addition of extract (1% of the medium) (E), and on the GO nanofilm with the addition of extract (1% of the medium) (GO-E). Passages were not performed for the experimental primary cell cultures. The medium was changed every 2 days.

The actual concentration of CEME added to the culture media was evaluated using Trypan Blue assay (NanoEnTek, Massachusetts, MA, USA). Cells were maintained with 5, 2.5, and 1% CEME supplementation in standard DMEM. After 48 h of exposure to CEME, cells were detached with trypsin. Then a suspension of cells in DMEM (10 μL) was mixed with trypan blue solution (10 μL). A mixture of cells and stain (10 μL) was placed onto a cell counting slide and the total and live cell counts were evaluated using an EVE^TM^ automatic cell counter (NanoEnTek, Massachusetts, MA, USA). The results were compared to the control group that was maintained without the addition of CEME. 

### 4.4. Cell Morphology

Cell morphology was observed and recorded using a light optical inverted microscope (TL-LED, Leica Microsystems, Germany) and a Scanning Electron Microscope (SEM) (Quanta 200, FEI, Oregon, OR, USA). Cells were fixed in ice cold methanol for 10 min at 4 °C and stained with eosin/hematoxylin. For SEM imaging, cells were fixed after 5 days of primary cell culture with 2.5% glutaraldehyde in phosphate-buffered saline (PBS) (Life Technologies, Texas, TX, USA). The samples were contrasted with osmium tetroxide (Sigma-Aldrich, Missouri, MO, USA) and carbohydrazide (Sigma-Aldrich, Missouri, MO, USA). The cells were then dehydrated in hexylene glycol (Sigma-Aldrich, Missouri, MO, USA); drying was performed using a Polaron CPD 750l critical point dryer (Quorum Technologies, Laughton, UK). 

The spontaneous contractions of muscle cells were observed and counted manually from randomly selected visual fields (*n* = 10) in real time (t = 1 min each series) using a light optical inverted microscope (TL-LED, Leica Microsystems, Germany). Time-lapse of contractions were performed on a confocal microscope (Olympus FV1000, Tokyo, Japan) with Nomarski contrast. Frames were captured every 6 s for 15 min. Supplementary Video S1 was speeded up 300 times. 

### 4.5. Cell Differentiation

The differentiation of cells was evaluated using a confocal microscope (Olympus FV1000, Tokyo, Japan). For imaging, cells were fixed after 5 days of culture using 4% paraformaldehyde in PBS (Life Technologies, Texas, TX, USA) for 10 min at room temperature and washed with ice-cold PBS. Then cells were incubated in Triton X-100 (Sigma-Aldrich, Missouri, MO, USA) and washed with PBS (Sigma-Aldrich, Missouri, MO, USA). Subsequently, cells were incubated with blocking solution, i.e., normal goat serum (Chemicon International, California, CA, USA). Cell nuclei were stained with 4′,6-diamidino-2-phenylindole (DAPI) (Thermo Fisher Scientific, Massachusetts, MA, USA) and actin filaments with phalloidin-Atto 633 (Sigma-Aldrich, Missouri, MO USA). After staining, the cells were washed with PBS. The fusion index was calculated for the control group and cells with CEME supplementation from randomly selected visual fields (*n* = 7) and presented as the number of nuclei in the multinucleated myotubes divided by the total number of nuclei. The nuclei were counted manually. 

### 4.6. LDH Assay

Cytotoxicity was evaluated using Cytotoxicity Detection Kit (Roche Diagnostics GmbH, Mannheim, Germany) after 48 and 96 h of primary cell culture. For the GO and GO-E groups, 40 μL of GO solution was dropped onto the bottom of the wells of 96-well microplates, dried, and incubated for 24 h with DMEM before starting cell culture. Cells were cultured in 96-well plates (approximately 10 × 10^3^ cells per well for a test after 48 h, and 5 × 10^3^ cells per well for a test after 96 h). The assay was performed according to the manufacture’s protocol. The absorbance of samples (*n* = 8) was measured at 450 nm using a Tecan Infinite 200 microplate reader (Tecan, Durham, NC, USA). The results were expressed as a relative value (related to the control) after subtracting the absorbance from blank samples. 

### 4.7. MTT Assay

Viability and proliferation were tested with a Vybrant Cell Proliferation Assay Kit (MTT) (Thermo Fisher Scientific, Massachusetts, MA, USA) after 48 and 96 h of primary cell culture. For the GO and GO-E groups, 40 μL of GO solution was dropped onto the bottom of the wells of 96-well microplates, dried, and incubated for 24 h with DMEM before starting cell culture. Cells were cultured in 96-well plates (as described above). MTT reagent (10 μL) was added to each well and incubated for 3 h at 37 °C. Next, lysis buffer (containing Isopropanol, Triton X, and HCl) was added to each well (100 μL). Culture plates were centrifuged, and the supernatant was transferred to new 96-well plates. The absorbance of samples (*n* = 8) was measured using Tecan Infinite 200 plate reader (Tecan, North Carolina, ND, USA) at 570 nm, according to the manufacturer’s protocol.

### 4.8. Gene Expression

Gene expression at the mRNA level was measured using the quantitative polymerase chain reaction method. The expression of *ATP5B*, *FGF2*, *LDH5*, *PCNA*, *Myf5*, *MyoD1*, *MyoG*, *Pax3*, and *Pax7* was examined, for calculations the *GAPDH* and *ACTB* housekeeping genes were used. All used primers are presented in Table 2. After 5 days of primary cell culture, cells were detached with trypsin and centrifuged at 1 200 rpm for 5 min. The cell pellet was homogenized using a TissueLyser ball mill (Qiagen, Germantown, IL, USA). Total RNA was isolated using a NucleoSpin (Marcherey, Nagel, Duren, Germany). Then, cDNA was synthesized with a Maxima First Strand cDNA Synthesis Kit for RT-qPCR (Thermo Scientific, Wilmington, DE, USA). The Real-Time PCR (RT-PCR) was carried out using a PowerUp SYBR Green Master Mix (Applied Biosystems, Michigan, MI, USA). The 2^-ΔΔCT^ method was used to determine the results.

### 4.9. Statistics

Data were analyzed using two-factorial and monofactorial analysis of variance, ANOVA, and StatGraphics Centurion version XVI (StatPoint Technologies, Georgia, GA, USA). Differences between groups were tested with Tukey’s honest significant difference post hoc test. There were significant differences between groups marked with different letters (a-b-c) and there were no significant differences between groups marked with the same letters (a-a; b-b; c-c). The level of significance was set at *p* ≤ 0.05. 

## 5. Conclusions

The GO nanofilm culture surface did not positively affect the differentiation or functional maturity of differentiated muscle fibers; however, it did promote spontaneous contractions. The chicken embryo muscle extract, collected on day 18 of embryonic development, contained a cocktail protein that influenced the intense differentiation of the muscle precursor cells collected on day 8 of embryo development. Compared to the cells in the control group, a structure resembling a pseudo-tissue was capable of spontaneous contractions, without the application of an electrical impulse. The addition of chicken embryo muscle extract, to a concentration of about 1% of culture medium, may allow researchers to culture pseudo-tissue muscle capable of spontaneous contraction. The study showed that GO may be used as a niche for differentiating muscle cells, but the decisive influence on the maturation of muscle tissue, especially muscle contractions, depends on the complexity of the applied growth factors.

## Figures and Tables

**Table 1:** Selected top proteins (59) from chicken embryo muscle extract specific for muscle cell activity.

Gene Name	Protein Name	Molecular Weight [kDa]
Extracellular matrix component		
DCN	Decorin	61.2
LAMB1	Laminin subunit beta-1	59.4
COL6A2	Collagen alpha-2 (VI) chain	58.7
A0A1D5PME9	Leucine rich repeat containing 15	50.3
FMOD	Fibromodulin	44.7
OGN	Mimecan/Osteoglycin	42.8
A0A1D5PVT6	Collagen type XI alpha 1 chain	36.4
LMNB1	Lamin-B1	31.6
PLOD1	Procollagen-lysine,2-oxoglutarate 5-dioxygenase 1	22.0
COL1A1	Collagen alpha-1 (I) chain	16.1
COL6A3	Collagen alpha-3 (VI) chain	14.6
LOC107050758	Collagen alpha-1 (II) chain	13.1
COL14A1	Collagen alpha-1 (XIV) chain	11.2
LABM1	Laminin subunit beta-1	10.4
Cell structure and communication		
PXN	Paxillin	66.6
P09652	Tubulin beta-4 chain	61.7
PTK7	Inactive tyrosine-protein	53.7
MAPT	Microtubule-associated protein	51.3
CRYAB	Alpha-crystallin B chain	50.3
MAPRE2	Microtubule-associated protein RP/EB family member 2	41.7
CDH13	Cadherin-13	40.4
COTL1	ADF actin binding protein	36.9
DMD	Dystrophin	24.1
ZYX	Zyxin	20.5
WIPF1	WAS/WASL interacting protein family member 1	18.8
CTNNA2	Catenin alpha-2	18.4
	Tubulin alpha chain	13.0
NHLRC2	NHL repeat-containing protein 2	12.4
CAP2	Adenylyl cyclase-associated protein	11.7
ACTG1	Actin, cytoplasmic 2	11.7
SPTB	Spectrin beta chain	10.4
JUP	Plakoglobin	9.92
DBN1	Drebrin	9.37
ACTN2	Alpha-actinin-2	8.02
Contractile apparatus		
MYL3	Myosin light chain	62.2
CALD1	Caldesmon	61.4
CAMK2D	Calcium/calmodulin-dependent protein kinase type II delta chain	55.3
CNN3	Calponin	51.3
MYLK	Myosin light chain kinase, smooth muscle	40.5
MYLPF	Myosin regulatory light chain 2, skeletal muscle isoform	37.0
TPM1	Tropomyosin alpha-1 chain	20.7
CASQ2	Calsequestrin	19.5
TNNC2	Troponin C, skeletal muscle	19.3
MYH1B	Myosin-1B	11.4
Neural and neuromuscular communication		
NEFM	Neurofilament medium polypeptide	46.2
AGRN	Agrin	46.1
TXLNB	Beta-taxilin	31.8
FABP5	Fatty acid binding protein 5	23.4
GAP43	Neuromodulin	18.8
NCAM1	Neural cell adhesion molecule	15.1
Metabolism		
ATP5C1	ATP synthase subunit gamma	53.3
GMPR	GMP reductase	49.9
GPD2	Glycerol-3-phosphate dehydrogenase	45.5
PFKM	ATP-dependent 6-phosphofructokinase	43.8
ADSSL1	Adenylosuccinate synthetase isozyme 1	43.6
CKM	Creatine kinase M-type	43.3
AMPD1	AMP deaminase	25.2
A0A1D5PIQ5	Mitogen-activated protein kinase	11.4
CKB	Creatine kinase B-type	5.62

**Table 2:** Primers for gene expression analysis using RT-qPCR.

Genes	Sequences (5′-3′)
*PCNA*	forward: TGCACGCATTTGTAGAGACC
	reverse: AGTCAGCTGGACTGGCTCAT
*FGF2*	forward: GCACTGAAATGTGCAACAG
	reverse: TCCAGGTCCAGTTTTTGGTC
*LDH-5*	forward: ATGCCCACAACAAGATCAG
	reverse: CCTTTCAGCTTGTCCTCCAC
*ATP5B*	forward: GTTATTCGGTGTTCGCTGGT
	reverse: TAGACCAGAGCGACCTTGG
*Myf5*	forward: CCAGGAGCTCTTGAGGGAAC
	reverse: AGTCCGCCATCACATCGGAG
*MyoD*	forward: GCTCTCGCAGGAGAAACAG
	reverse: CTGGAGGCAGTATGGGACAT
*MyoG*	forward: GCTGAAGAAGGTGAACGAA
	reverse: CTGCTGGTTGAGGCTGCT
*Pax3*	forward: CCGTGCTAGATGGAGGAAGC
	reverse: AGACACGGCTTGCGGTATG
*Pax7*	forward: CAGTAGAGACAGGCCAAGC
	reverse: GGAGTTGGGAAGGAGTAGGG
*GAPDH*	forward: GAGGACCAGGTTGTCTCCTG
	reverse: CCACAACACGGTTGCTGTAT
*ACTB*	forward: GTCCACCTTCCAGCAGATGT
	reverse: ATAAAGCCATGCCAATCTCG

## Supplementary Materials

The following are available online, Supplementary Table S1: The list of identified protein in chicken embryo muscle extract (CEME) by LC-MS. Video S1: Spontaneous contraction activity of cells with chicken embryo muscle extract (CEME) supplementation, 1 to 3 contractions per minute were noted, average 1.7 contraction per minute.
